# Age- and Concentration-Dependent Elimination Half-Life of 2,3,7,8-Tetrachlorodibenzo-*p*-dioxin in Seveso Children

**DOI:** 10.1289/ehp.8884

**Published:** 2006-07-06

**Authors:** Brent D. Kerger, Hon-Wing Leung, Paul Scott, Dennis J. Paustenbach, Larry L. Needham, Donald G. Patterson, Pier M. Gerthoux, Paolo Mocarelli

**Affiliations:** 1 Health Science Resource Integration, Tallahassee, Florida, USA; 2 Private Consultant, Danbury, Connecticut, USA; 3 ChemRisk, San Francisco, California, USA; 4 Centers for Disease Control and Prevention, Atlanta, Georgia, USA; 5 Department of Laboratory Medicine, University Milano-Bicocca, Hospital of Desio, Desio–Milano, Italy

**Keywords:** children, dioxin, elimination, half-life, model, pharmacokinetics

## Abstract

**Objective:**

Pharmacokinetic and statistical analyses are reported to elucidate key variables affecting 2,3,7,8-tetrachlorodibenzo-*p*-dioxin (TCDD) elimination in children and adolescents.

**Design:**

We used blood concentrations to calculate TCDD elimination half-life. Variables examined by statistical analysis include age, latency from exposure, sex, TCDD concentration and quantity in the body, severity of chloracne response, body mass index, and body fat mass.

**Participants:**

Blood was collected from 1976 to 1993 from residents of Seveso, Italy, who were < 18 years of age at the time of a nearby trichlorophenol reactor explosion in July 1976.

**Results:**

TCDD half-life in persons < 18 years of age averaged 1.6 years while those ≥18 years of age averaged 3.2 years. Half-life is strongly associated with age, showing a cohort average increase of 0.12 year half-life per year of age or time since exposure. A significant concentration-dependency is also identified, showing shorter half-lives for TCDD concentrations > 400 ppt for children < 12 years of age and 700 ppt when including adults. Moderate correlations are also observed between half-life and body mass index, body fat mass, TCDD mass, and chloracne response.

**Conclusions:**

Children and adolescents have shorter TCDD half-lives and a slower rate of increase in half-life than adults, and this effect is augmented at higher body burdens.

**Relevance:**

Modeling of TCDD blood concentrations or body burden in humans should take into account the markedly shorter elimination half-life observed in children and adolescents and concentration-dependent effects observed in persons > 400–700 ppt.

Shorter elimination half-lives for 2,3,7,8-tetrachlorodibenzo-*p*-dioxin (TCDD) and other polychlorinated dibenzo-*p*-dioxins and polychlorinated dibenzofurans (PCDD/Fs) have been reported in human infants (Kreuzer et al. 1997; Leung et al. 2006) and in highly exposed adults (Aylward et al. 2005; Leung et al. 2005) compared with those in the general population. However, few published elimination half-life data are available for young children and adolescents 1–18 years of age. Needham et al. (1997/1998) presented TCDD decay curves for a 50-year-old Seveso male (initial TCDD concentration of 1,770 ppt) and a 6-year-old Seveso male (initial TCDD concentration of 15,900 ppt) and noted a much faster TCDD serum lipid decay for the child, especially over the 6-year period following exposure. Additional data on children are needed to further validate the two age-dependent PCDD/F half-life models that have been proposed for estimating childhood body burdens (Kerger et al. 2005; Kreuzer et al. 1997).

In this study we examined a database of longitudinal TCDD measurements in the blood lipids of children (ages 0.5–18 years) exposed during the 1976 trichlorophenol reactor explosion incident near Seveso, Italy. As many as 10 sequential measurements were made on some children. We evaluated changes in elimination rate of TCDD in blood lipids as influenced by age, latency from exposure, TCDD concentration or mass in the body, severity of chloracne, and body mass parameters potentially influencing the half-life in children, adolescents, and young adults. Our goal was to identify central tendency trends for the half-life versus age relationship that may be used to estimate childhood body burdens, particularly for children 1–7 years of age, an age range critical to understanding potential risks of PCDD/F intake during childhood (Kerger et al. 2005).

## Materials and Methods

Data from the Seveso incident include fairly complete information on longitudinal blood TCDD measurements, sampling date, exposure zone, severity of chloracne, and age, height, and weight at the time of sampling (up to 16 years after the incident). Persons < 18 years of age in July 1976 with at least two blood TCDD measurements were included in the initial data evaluation, which comprised 27 females and 20 males within Zone A. The analytical method for lipid TCDD has been reported by Patterson et al. (1987), and some clinical correlations have been reported by Mocarelli et al. (1991).

The calculated half-life of TCDD was based on one or more data pairs of serum lipid TCDD concentrations for each individual. The initial peak TCDD concentration in several individuals occurred 3–12 months after July 1976, indicating continuing absorption or redistribution among tissues. The elimination half-life was calculated using the standard equation as described by Galbaldi and Perrier (1982):


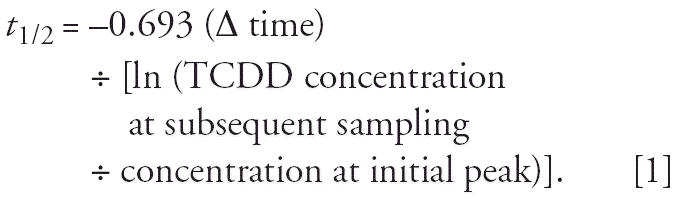


The peak TCDD measurement between July 1976 and July 1977 served as the initial value (A) in chronological sequence (A→B→C→D), and data pairs (A–B, A–C, A–D) were used to calculate half-life by the foregoing equation. The initial value (A) is referred to as the initial sample and the samples collected after A (B, C, and D) are referred to as the subsequent samples. Undetected values were included in the analysis at the stated detection limit.

Some of the analyses used estimated values for body mass index (BMI) and body fat mass (BFM) relevant to the sampling time and individual. BMI was calculated using measurements of the metric height (H) in centimeters and weight (W) in kilograms and the standard equation:


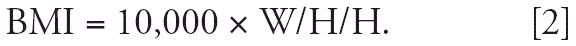


We calculated BFM by estimating the body fat fraction correlated to BMI with age- and sex-specific equations reported by Deurenberg et al. (1991) and multiplying by body weight to obtain total kilograms of body fat. TCDD mass in the body fat (in nanograms) was calculated by multiplying BFM (in kilograms) and TCDD concentration (in parts per trillion, or nanograms TCDD per kilogram lipid) at the time of blood sampling.

Preliminary analysis of data correlations indicated the expected data scatter from typical laboratory analytical error (e.g., ± 10–30%) plus more substantial outliers that skewed the central tendency trends. Many of the extreme high and low half-life values were seen in the first year of measurements. These likely represent additional environmental exposures and/ or slow equilibration of the body TCDD dose, both of which would make half-life calculations less reliable for that period. Thus, all data pairs occurring before July 1977 were excluded (nine positive half-lives excluded: 0.1, 0.2, 0.3, 0.4, 0.6, 0.6, 0.8, 7.4, and 23.8 years). In other cases, outlier values occurred as unusually high or low measurements within the first 5 years of data (through 1981). We excluded data pairs in this time period only if the half-life was > 2-fold higher or lower than the median of the values for that individual and/or for others within a ± 3-year age span (eight positive half-lives excluded: 0.7, 7, 8.1, 14.8, 18.5, 19.4, 27.9, and 30.7 years).

We analyzed selected data groupings by linear regression and Student’s *t*-test using the algorithms in Microsoft Excel 2000 or using the *Z*-statistic test (Freund 1970) for comparing means among groups of greatly different sample sizes. Selected subsets were defined according to age, body fat parameters, chloracne grade/status, and sex. Each data grouping was evaluated for age-dependent and concentration-dependent effects on TCDD elimination half-life.

We evaluated relationships between TCDD half-life and several independent variables that describe age, body composition, and TCDD body burden using a series of mixed regression models [see Supplemental Material (http://www.ehponline.org/members/2006/8884/supplemental.pdf)]. Due to an observed TCDD concentration-dependent effect on half-life with a transition point around 700 ppt, TCDD concentration was included as a categorical variable in a mixed regression analysis reflecting the slopes above and below the transition point. TCDD concentrations ≤700 ppt were given a value of zero and concentrations > 700 ppt were given a value of 1. Also, an interaction term, age × concentration, was included to evaluate the effect of concentration on the slope of the age versus half-life relationship.

## Results

**Figure 1:**
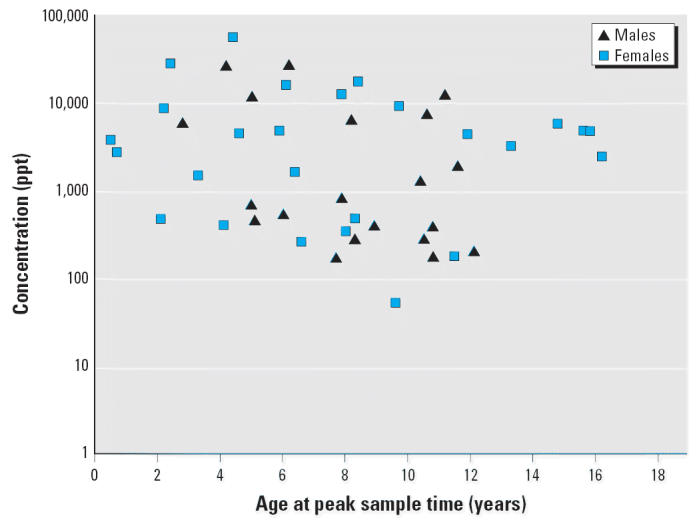
Peak TCDD concentration versus ages of Seveso children. Highest reported blood lipid TCDD concentrations for 27 female and 20 male Seveso residents who were < 18 years of age in 1976 and had at least two valid samples.

Blood TCDD measurements analyzed here include only those Seveso residents in Zone A who were < 18 years of age in July 1976 and had at least two measurements; this included 27 females and 20 males. The age and peak TCDD concentration for each subject are plotted in Figure 1. At the first sampling in 1976, the males ranged from 2.8 to 12.1 years of age and had peak TCDD levels from 173 to 26,400 ppt. The females ranged in age from 0.5 to 16.6 and had peak TCDD levels from 54 to 56,000 ppt. Two females with limited data were excluded. One female had only two samples taken (at 0.5 and 0.8 years of age, both in 1976) and peak TCDD of 3,770 ppt, and was excluded because of the less-than-1-year-since-exposure criterion. The second female was sampled at 8–11.8 years of age; she had a peak TCDD level of 339 ppt and was excluded on the basis of an outlier half-life value (30.7 years). Thus, the final cohort is comprised of 25 females and 20 males who each contributed at least one data pair for the half-life trend analyses.

**Figure 2:**
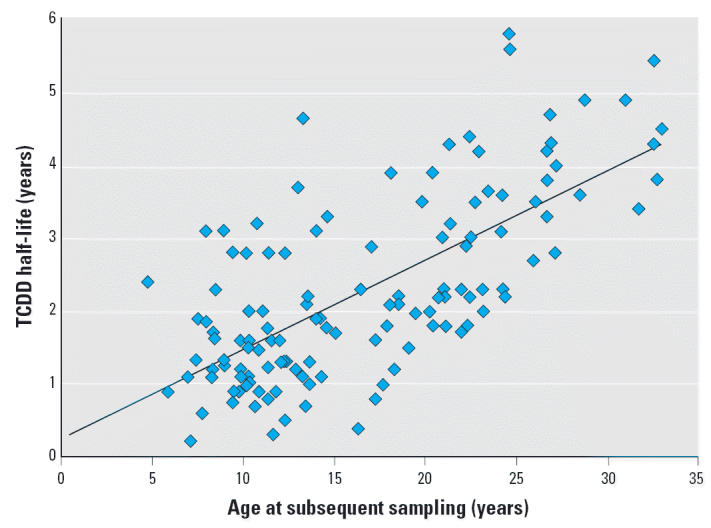
TCDD half-life versus age of Seveso children. Linear regression analysis of entire cohort (25 females, 20 males) with the half-life since peak TCDD measurement plotted against the person’s age at time of subsequent sampling. See “Materials and Methods” for outliers identified and excluded. See also Table 1.

The correlation between TCDD half-life and age for the final cohort is given in Figure 2, showing a direct linear relationship with a mean slope of 0.12 (95% confidence interval, 0.10–0.14) and a moderate correlation coefficient of 0.48. As illustrated in Table 1, this linear correlation between TCDD half-life and age is dominated by measurements collected after the individuals reached 18 years of age, with flatter slopes and poor correlation coefficients obtained for measurements before age 18. Similar trends were identified for subgroups selected on the basis of latency since the exposure incident, although correlation coefficients were stronger for age-dependency (Table 1). Unfortunately, insufficient data are available for individuals < 12 years of age to derive a meaningful regression analysis (*r*^2^ < 0.01). However, the available data indicate a more gradual or flatter slope for the youngest members in this cohort.

Table 1 presents a series of selected mean comparisons and linear regression of the TCDD half-life versus age relationship. Statistically significant influences (*p* < 0.05) on average half-life were observed for BMI, TCDD concentration (in subsequent blood samples), TCDD mass in the body fat, chloracne presence/severity, age, and latency. Sex appeared to have a minor influence on half-life, but the average half-life for males is not significantly different than for females (*p* > 0.05), although Needham et al. (1994) reported a longer serum TCDD half-life in adult women compared with adult men.

With respect to BMI, heavier individuals (who inherently have more adult measurements) had significantly higher mean half-lives than leaner individuals in paired comparisons above and below BMIs of 20 and 25 (Table 1). However, the linear regression slope of half-life versus age did not show consistent trends when comparing the leaner and heavier BMI groups in Table 1. Notably, there were considerable differences in average age between the leaner and heavier BMI groups that may have influenced the correlation. Also, data pairs for two extremely obese females (17 years of age, BMI 36, half-life 2.9 years; and 22 years of age, BMI 39.2, half-life 1.7 years) were excluded from the linear regression analyses for BMI > 20 because they substantially skewed the slope (from 0.11 to 0.08) and correlation (*r*^2^ from 0.42 to 0.05); however, their inclusion did not alter the mean half-life comparison.

A concentration-dependent effect appears to coincide with the age-dependent effect on half-life in children and adolescents (< 18 years of age). This is indicated by significantly shorter mean half-life at higher blood lipid TCDD concentrations (> 700 ppt) and higher TCDD mass in the body fat (> 7,000 ng) compared to means for the paired, lower body burden groups (Table 1). Subgroups selected on the basis of these higher body burdens demonstrated flatter and more poorly correlated slopes for half-life versus age, although these groups are less robust in terms of the number of individuals and samples represented. In contrast, the lower body burden groups demonstrated slopes and correlation coefficients consistent with the whole cohort analysis.

Subgroups selected according to chloracne response showed significantly shorter mean half-lives with increasing severity of chloracne compared with the subgroup with no chloracne (Table 1). No statistically significant sex-related differences were identified, although males consistently showed a slightly longer average half-life than females in paired comparisons (Table 1).

**Figure 3:**
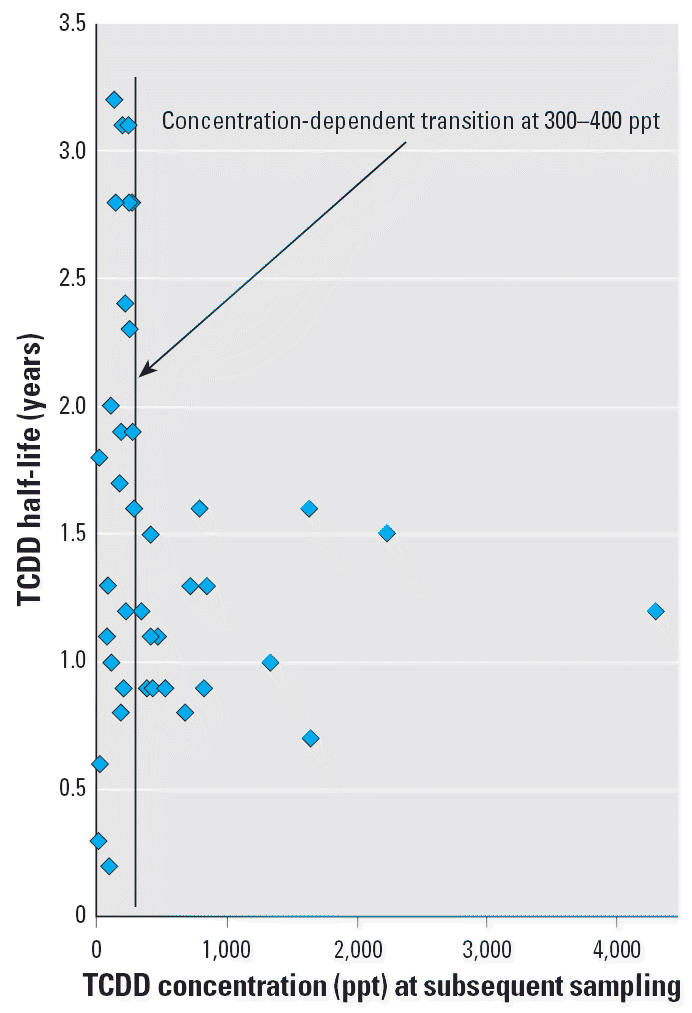
TCDD half-life versus subsequent blood lipid TCDD concentration for Seveso children < 12 years of age. Data subset excluding all measurements in persons ≥12 years of age, including 13 females and 9 males (43 values). TCDD concentrations above the transition range of 300–400 ppt correspond to uniformly shorter half-lives. Similar trends were apparent when all ages were included, with an observed transition of approximately 700 ppt. See also Table 1.

Overlapping age- and concentration-dependent trends are inherent to the selected cohort in that most of the individuals with relatively high peak TCDD levels (i.e., 26 of 29 persons > 1,000 ppt) were ≤12 years of age at the time of the reactor explosion (Figure 1). Table 1 shows similar average half-lives for persons < 12 and < 18 years of age, and a significantly longer half-life for persons > 18 years of age. Similarly, persons with > 15 years of latency since exposure showed significantly longer average half-life compared to persons with < 15 years latency (Table 1). Figure 3 presents the TCDD half-life vs. blood lipid concentration relationship for children < 12 years of age [i.e., data pairs for higher ages were excluded; and TCDD values are the subsequent (not peak) concentrations]. An apparent transition range is seen for shorter half-lives around 300–400 ppt, with the longest half-life in males at 1.2 years and in females at 1.7 years for TCDD concentration > 400 ppt. Similar analysis of the entire cohort showed no half-lives > 2 years in those with > 2,000 ppt, and only 2 half-life values > 2.2 years at > 700 ppt (data not shown).

Overall, linear regressions of half-life versus age in Table 1 show consistent slopes of 0.1–0.15 for the entire cohort and in the more robust subgroups except those selected according to age, latency, or TCDD concentration > 700 ppt. The 95% confidence intervals (CIs) for all linear regression slopes in Table 1 overlapped and were not significantly different (*p* > 0.05; data not shown). Many of the significant differences between mean half-lives may be associated with inadvertent age differences among the selected subgroups.

Simple linear regression analysis revealed relationships between body composition, age, and TCDD half-life. Moderate correlations were found for BFM vs. age [Equation 3], BMI vs. age [Equation 4], half-life vs. BFM [Equation 5], and half-life vs. BMI [Equation 6]:


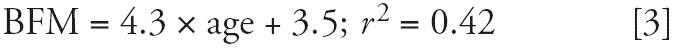



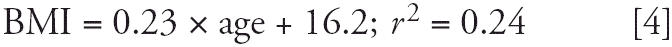



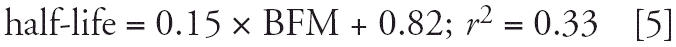



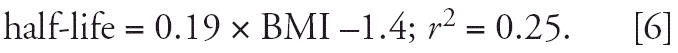


The mixed regression model that included age, TCDD concentration category, and TCDD concentration category × age indicated that the subject effect was not statistically significant (*p* = 0.6). Accordingly, we used a backward stepwise regression procedure to identify the most appropriate multiple regression model based on a starting model that included age, TCDD concentration category, and TCDD concentration × age.

After accounting for the TCDD concentration × age term’s effect on the slope of age, the final model for TCDD concentration 700 ppt is


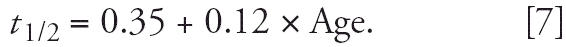


For TCDD concentration > 700 ppt the final model is


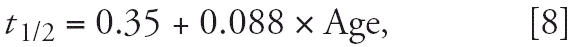


where *t*_1/2_ is the half-life and Age is the age at time of subsequent sampling. The final model included age and TCDD concentration category × age with an *r*^2^ of 0.71. The coefficients of both age (*p* < 0.0001) and TCDD concentration × age (*p* = 0.028) were significantly different than zero. This model indicates that increased TCDD concentrations affect the rate at which half-life increases with age rather than the baseline half-life associated with an age of 0 years. For two children with the same age but TCDD concentrations below or above 700 ppt, the child with a TCDD concentration < 700 ppt will have, on average, a longer half-life than the child with a TCDD concentration > 700 ppt.

**Figure 4:**
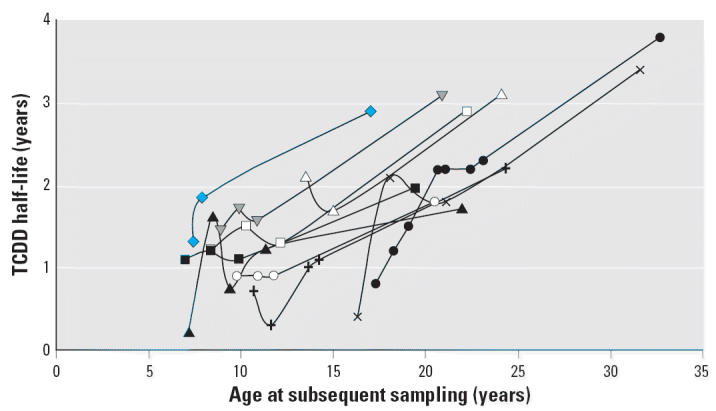
Individual TCDD half-life versus age for peak TCDD > 2,000 ppt. Subset includes only persons with ≥3 valid half-lives and peak blood lipid TCDD concentration > 2,000 ppt. Each symbol represents an individual with at least three half-life estimates. Linear regression was performed by individual showing parallel slopes (mean ± SD 0.12 ± 0.036 year/year) and 7 of 10 individuals with *r*^2^ ≥0.91 (data not shown). Similar trends were found for 7 individuals with 3 or more valid half-lives and peak TCDD < 2000 ppt, showing parallel slopes (0.14 ± 0.062 year/year) and 5 of 7 individuals with *r*^2^ ≥0.88 (data not shown).

The trends of individual persons for TCDD half-life versus age (at subsequent sampling) among those with ≥3 valid data pairs and higher TCDD concentrations (> 2,000 ppt at peak) show parallel slopes (Figure 4). Linear regression by individual results in an average slope of 0.12 ± 0.036 year/year (mean ± SD; *n* = 10), consistent with overall data trends in Figure 2. Similar analysis of individuals with lower TCDD blood concentrations (< 2,000 ppt) shows an average slope of 0.14 ± 0.06 year/year (*n* = 7; data not shown). Thus, lower blood concentrations seem to correspond to slightly higher slope, although the difference is small and not statistically different (*p* > 0.05).

**Figure 5:**
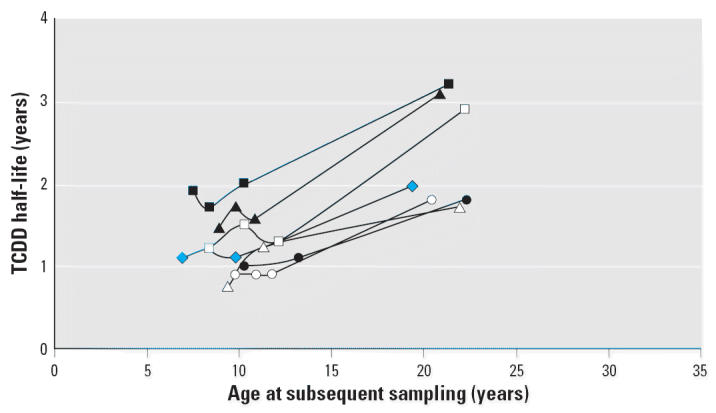
Individual trends for TCDD half-life versus age for peak exposure at < 7 years of age. Subset includes only persons with ≥3 valid half-lives and < 7 years of age at time of peak blood lipid TCDD concentration. Each symbol represents an individual with at least three half-life estimates. Linear regression was performed by individual showing parallel slopes (mean ± SD 0.09 ± 0.027 year/year) and 6 of 7 individuals with *r*^2^ ≥0.93 (data not shown).

Individual person trends for TCDD half-life versus age among those with three or more valid data pairs and first exposure before 7 years of age also show parallel slopes (Figure 5). Linear regression by individual shows a mean (± SD) slope of 0.09 ± 0.027 year/year (*n* = 7). Thus, younger age at first exposure seems to correspond to lower slope. This is consistent with the flatter slope observed in persons < 18 years of age for the total cohort in Table 1.

**Figure 6:**
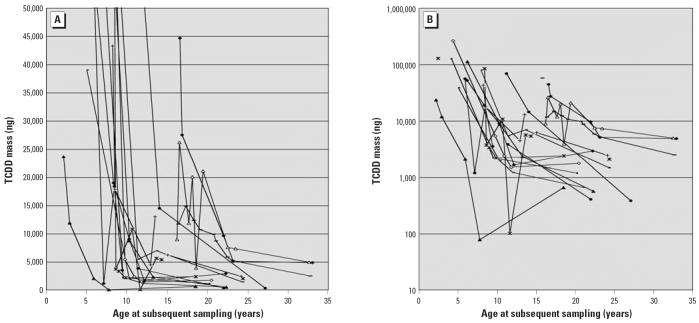
TCDD Mass in the body fat versus age of Seveso children for peak TCDD mass > 10,000 ng. Subset including only persons with ≥3 valid half-lives and peak TCDD mass > 10,000 ng. Each symbol represents an individual with at least three TCDD mass determinations. (*A*) TCDD mass in linear scale; TCDD mass = 50,000 ng to help visualize trends apparent < 10,000 ng at time of sampling. (*B*) TCDD mass in log scale with no truncation of data. The estimated transition point for shorter TCDD half-life is > 7,000 ng. See also Table 1.

Individual person trends for TCDD mass in body fat versus age among those with ≥3 valid data pairs and > 2,000 ppt peak TCDD concentration in blood lipid are shown in Figure 6. The TCDD mass parameter is already adjusted for individual differences in BMI and adipose volume. An initial phase of rapid and substantial loss of TCDD mass from the body is illustrated in Seveso children and adolescents < 18 years of age and/or with body burdens above approximately 7,000 ng (about 500 ppt in a 70-kg person with 20% body fat), with transition to a more gradual slope of half-life versus age for those > 18 years of age and < 7,000 ng (Figure 6, Table 1).

## Discussion

Few published data are available on TCDD elimination half-life in humans between the ages of 1 and 18 years. This study shows a dominant and consistent influence of age on TCDD half-life for this cohort of 45 Seveso residents exposed as children and adolescents in 1976. Mean TCDD half-life for persons < 18 years of age (or with < 15-year latency) was about half that for persons > 18 years of age (or with > 15-year latency). The slope of the half-life versus age plot averages 0.12 (95% confidence interval, 0.10–0.14) years increased TCDD half-life for each year of age. Age probably explains most of the variance in the individual half-life trends, but is co-correlated with TCDD concentration, BMI, and body fat mass. Children < 12 years of age and/or first exposed < 7 years of age showed flatter slopes for the rate of increase in TCDD half-life with age compared to the > 18-year age group.

Higher peak blood lipid TCDD concentrations (> 400 ppt in children and > 700 ppt in adults, or > 7,000 ng TCDD mass in the body fat) were associated with shorter mean TCDD half-life and a reduced slope of the half-life versus age correlation; that is, a greater excretion rate was observed for the most highly exposed Seveso children. These findings are consistent with those of Aylward et al. (2005) who examined age- and concentration-dependent half-life trends among Seveso adults and in a small group of Austrian patients (Emond et al. 2004; Geusau et al. 2002). Aylward et al. (2005) reported that TCDD body burdens > 10,000 ppt in blood lipid corresponded to half-lives < 3 years in adults (e.g., 20–50 years of age). This study identifies even shorter TCDD half-lives for children and adolescents, averaging 1.3 years for females and 2.0 years for males < 18 years of age, and showing a steady rate of increase in half-life with age. Using the final regression model for the Seveso child cohort (defining half-lives above and below the 700 ppt transition point) that includes age at time of sampling and TCDD concentration times age, a 21-year-old person with a background TCDD level of 10 ppt in serum lipids would exhibit a half-life of 2.9 years, and the same person with a high TCDD body burden of 100,000 ppt would exhibit a half-life of 2 years. By comparison, a child with comparable TCDD body burdens at 7 years of age would exhibit a TCDD half-life of 1.2 years at background exposure levels and 0.9 years at 100,000 ppt. The simple models for calculating TCDD half-life are based primarily on measurements spanning the range from childhood and early adolescence to early adulthood. Models based primarily on adult data (Aylward et al. 2005) may be more appropriate for predicting age- and concentration-dependent half-life for older adults.

Because adipose tissue volume increases with age, the age-dependency of TCDD half-life co-correlated (as expected) with parameters generally reflecting adipose tissue volume. Leaner body mass (BMI < 20) was associated with shorter average half-life, whereas higher BFM or BMI were generally associated with a longer average half-life; however, the opposite trend was seen in two extremely obese females (BMI > 35, 17 and 22 years of age). One might expect that the shorter half-life in leaner subjects may reflect a higher TCDD concentration gradient favoring excretion via fecal lipids (Schlummer et al. 1998). The two clinically obese females each demonstrated depuration of most of their TCDD body burden before becoming obese. The 17-year-old subject with a BMI of 36 had a peak TCDD mass in body fat of 7,600 ng at 1.7 years of age, which was reduced to 2,200 ng by 7.9 years of age, when her BMI was 22.7. The next blood measurement at age 17 when clinical obesity was noted corresponded to a TCDD mass of 2,600 ng, showing little change in the 9 years since the previous measurement. The 22-year-old subject with a BMI of 39 had a peak TCDD mass of about 56,000 ng at 5.9 years of age, which was reduced to 3,900 ng at 11.4 years of age, when her BMI was 23.7. The next blood measurement, at 22 years of age, when clinical obesity was present, corresponded to a TCDD mass of only 410 ng, far lower than the body burden about 10 years earlier. Thus, both clinically obese subjects had apparently excreted most of their TCDD body burden before becoming obese. Severe obesity was apparently associated with total sequestration (full retention) of the TCDD body burden in a subject 17 years of age, with moderate initial body burden (about 2,800 ppt in serum lipid and 7,600 ng TCDD in the body) and with continued elimination in a second subject, 22 years of age, with a higher initial body burden (about 4,800 ppt in serum lipid and 56,000 ng TCDD in the body). Although this deserves further study, the number of overweight and obese subjects in this child exposure cohort seems too small to provide meaningful insights.

In more detailed mixed regression modeling, the half-life in this population was significantly influenced by variables reflecting time (age at subsequent sampling or initial age combined with latency at subsequent sampling) and TCDD body burden (initial concentration, subsequent concentration, and mass in the body); however, age-related parameters explained most of the variance. BFM was a significant contributor only in models that excluded contributions from the age-related variables, but BMI was not a significant contributor in the mixed regression models [see Supplemental Material (http://www.ehponline.org/members/2006/8884/supplemental.pdf)]. Complex cross-correlation between age- and concentration-related effects on half-life may have masked the less predominant influence of body composition on TCDD half-life in this study population.

Consistent with various pharmacokinetic models based primarily on distribution of TCDD into adipose volume (Carrier et al. 1995a, 1995b; Kerger et al. 2005; Kreuzer et al. 1997), an existing TCDD body burden becomes sequestered in the larger/growing lipid volume, leading to apparently longer half-life with increasing age. However, growth of the adipose tissue compartment during childhood appears to explain only a small portion of the age-related changes in TCDD excretion based on the pattern of loss of TCDD mass in adipose tissue (Figure 6). Also, the inverse correlation between severity of chloracne and average half-life (Table 1) may reflect greater excretion via sebaceous glands and/or sequestration in the skin among those most highly exposed (Iida et al. 1997; Matsueda et al. 1995). Kerger and James (2006) estimated that sebaceous oil secretions and sloughing of skin epidermis might account for twice as much PCDD/F elimination as that related to fecal excretion alone. Enhanced biliary/fecal excretion and/or induction of liver binding proteins may also play a role at these higher doses (Carrier et al. 1995a, 1995b; Leung et al. 1990a, 1990b).

The observed simple linear regression of half-life versus age for the Seveso child cohort (Figure 2) shows a predicted TCDD half-life of 0.24 to 0.38 years at birth and 1 year of age, respectively. A predicted TCDD half-life of approximately 0.4 years in infants is consistent with the pharmacokinetic model predictions and observations of Kreuzer et al. (1997) and Leung et al. (2006) for infants with background body burdens of PCDD/Fs. These findings for TCDD half-life in Seveso children are also consistent with results reported by Leung et al. (2005) showing pentachlorinated dibenzofuran (PeCDF) and hexachlorinated dibenzofuran (HxCDF) half-lives in people (17–80 years of age) highly exposed during the Yusho and Yucheng poisoning incidents in Japan and Taiwan, respectively. The age-related increases in half-life reported for PeCDF and HxCDF (combined cohort trends of 0.18 and 0.12 year/year, respectively) are similar to those reported here for TCDD in the whole Seveso child cohort and in the more robust data subsets (0.11–0.15 year/ year). Leung et al (2005) also identified distinctly shorter half-lives for those individuals with the highest tissue concentrations (e.g. > 3,000 ppt), similar to that seen for the entire Seveso child cohort.

In this study we aimed to identify central tendency and more robust trends influencing TCDD half-life in Seveso children. Several caveats are evident from our analysis. First, several individuals were observed to have increasing blood lipid TCDD concentrations up to a year after the explosion event in July 1976, indicating further exposure and/or gradual equilibration of the TCDD dose with blood lipids. This finding indicates that the true peak TCDD level may have been missed for the many individuals with only one or two measurements in the first year. Missing the peak blood concentration would lead to higher-than-actual estimated half-life values. Second, as noted in “Materials and Methods,” we assumed that nondetect values were present at the stated detection limit. This would also have a tendency to overstate the half-life if the true TCDD concentration was lower. Third, a number of data pairs were identified that skewed trends considerably from the central tendency, which led us to exclude a comparable number of extremely low (e.g., < 0.6 year) and extremely high (e.g., > 10 year) half-life values. On balance, deleting these values reduced the variance but did not change the robust central tendency trends. Fourth, some individuals had many sequential measurements (e.g., 4–10 data pairs), whereas most had only a few (e.g., 1–2 data pairs). Also, the time span between sequential blood TCDD measurements was not uniform across subjects; the influence of this factor on individual or population half-life trends, if any, is unknown. Among individuals with ≥3 valid data pairs, 10 (3 males/7 females) had higher grade chloracne (grade 3–4), 3 (3 females) had lower grade chloracne (grade 1–2) and 9 (7 males/2 females) had no chloracne. Accordingly, the overall trends may overrepresent female subjects with chloracne and males without chloracne. And fifth, the data set did not contain a sufficiently large number of valid data pairs to examine each *a priori* variable thoroughly. Few observations were obtained for very young children (< 7 years of age) or for clinically obese individuals (BMI > 35). Thus, the findings based on less robust data should be interpreted with caution.

These findings may help validate the assumptions used in pharmacokinetic models that attempt to predict body burden trends for TCDD (Kreuzer et al. 1997) and all PCDD/Fs (Kerger et al. 2005) throughout the human lifespan. An important element of TCDD body burden modeling is to better understand the underlying reasons responsible for the decrease in lipid concentrations, which may be caused by dilution in a larger adipose compartment, to tissue-specific sequestration (e.g., to CYP1A2-binding proteins in the liver), and/or to true elimination of TCDD from the body. For example, Kreuzer et al. (1997) presented a pharmacokinetic model for TCDD that accounts for age-related dilution with growth of the adipose compartment, as well as contributions from losses related to metabolism and excretion by partitioning to fecal fats, although metabolism seems very limited in humans (Hu and Bunce 1999; Rohde et al. 1999).

The exact contribution of these factors is not known and can vary depending on circumstances, but the available evidence suggests that *a*) a lipid TCDD dilution effect from adipose growth and *b*) fecal lipid excretion each are likely to play a substantial role, with metabolism being a moderate or minor contributor. Excretion via fecal lipids may have a particularly important role for infants and young children, as their rate of fecal lipid excretion per body weight basis is about 7 times higher than that of adults (International Commission for Radiological Protection 1975). In highly exposed persons with maximal CYP1A2 induction, hepatic sequestration with subsequent redistribution of the PCDD/Fs from fat to the liver may be a dominant factor (Abraham et al. 2002; Grassman et al. 2000). And in persons with severe chloracne, loss via sebaceous lipids and skin desquamation may be appreciable. Our observation that the decline in the total mass of TCDD in Seveso child body fat (a metric that is normalized for the effects of adipose compartment growth) exceeds that plausibly caused by growth (Figure 6) suggests that excretion played an important role, particularly that during the first 5–10 years after the peak body burden.

With respect to children < 12 years of age, the available data indicate short PCDD/F half-lives for infants [0.4 years for TCDD at 0–1 years of age; based on data from Leung et al. (2006)] and a consistent rate of half-life increase with age based on the current study and other data (Leung et al. 2005). Although the half-life data on the 1- to 12-year age group are limited, a slower or flatter rate of increase compared to older adolescents and young adults is suggested in the current study. Therefore, use of the higher half-life versus age slope relevant to early adolescence through adulthood (e.g., 0.12 year/year) is suggested as a conservative yet appropriate assumption for modeling TCDD body burdens near background levels for younger children.

The shorter half-life of TCDD in children and adolescents may also be important with respect to assessing health risks from environmental exposures of potential concern. For example, dioxins present in breast milk and cow’s milk are of concern because of the higher intake rates relative to body size for young children (Agency for Toxic Susbstances and Disease Registry 1998). The shorter half-lives for TCDD in early childhood correspond to much lower tissue concentrations and presumably lower health risks during high intake periods of breast-feeding or higher cow’s milk intake, compared to predictions based on adult half-life assumptions (Kerger et al., in press; Kreuzer et al. 1997; Paustenbach et al. 2006; Richter et al. 2006). Similar implications apply for potential health risks from dioxin intake via contaminated soils, which are expected to be greater in younger children due in part to frequent mouthing behaviors under age 5 (U.S. Environmental Protection Agency 2000). Further, the shorter half-life for TCDD during early childhood and adolescence will inherently influence the body burdens in reproductive-age women, reflecting an attenuated influence of childhood exposures on body burdens transferred to offspring, both *in utero* and via lactation (Richter et al. 2006). Further research is warranted to assess these possible implications.

In summary, this study identifies significantly shorter TCDD half-life in children and adolescents compared to adults. Half-life is influenced predominantly by age and blood lipid TCDD concentration at the time of subsequent sampling, with linear equations providing good fit to the data above and below a transition range of 400–700 ppt. Increasing body mass index or body fat mass generally correspond to longer half-life, consistent with a sequestration effect on TCDD elimination as adipose volume grows. However, assessment of “TCDD mass in the body fat” indicates age- and concentration-dependent elimination trends that are largely independent of adipose volume. These pharmacokinetic considerations should be taken into account when evaluating TCDD dose–response relationships in epidemiologic studies and for risk assessment purposes.

## Figures and Tables

**Table 1:** Statistical analysis of means and linear regression trends for TCDD half life vs. age for selected subgroups of Seveso children.

Subgroup	*n*_female_ (*n*_values_)	*n*_male_ (*n*_values_)	Half-life (years) (mean ± SD)	Age (years) (mean ± SD)	Slope	Intercept	Correlation (*R*^2^)
BMI comparisons (mean ± SD)							
All (BMI = 20.2 ± 3.4)	25 (66)	20 (50)	2.4 ± 1.3	16.8 ± 7.1	0.12	0.18	0.48
With BMI < 20 (BMI = 16.9 ± 1.7)	16 (27)	9 (17)	1.8 ± 1.1	13.1 ± 5.5	0.15	0.10	0.51
With BMI > 20 (BMI = 22.9 ± 2.2)	17 (28)	18 (29)	2.8 ± 1.2^a^	19.1 ± 7.4	0.11	0.75	0.42
With BMI < 25 (BMI = 19.5 ± 2.9)	24 (55)	15 (37)	2.2 ± 1.1^a^	15.6 ± 6.9	0.11	0.44	0.45
With BMI > 25 (BMI = 28.3 ± 4.3)	3 (4)	9 (9)	3.9 ± 1.4^a^,^b^	24.6 ± 5.3	0.19	0.74	0.51
TCDD concentration comparisons							
With TCDD conc < 700 ppt (avg conc= 219 ± 179 ppt)	25 (58)	20 (51)	2.4 ± 1.3	17.1 ± 7.4	0.12	0.32	0.50
With TCDD conc > 700 ppt (avg conc = 1,400 ± 796 ppt)	10 (17)	3 (3)	1.6 ± 0.8^c^	13.9 ± 4.5	0.08	0.65	0.14
TCDD mass in the body comparisons							
With TCDD mass < 7,000 ng (avg mass = 2,042 ± 1,763 ng)	25 (52)	20 (44)	2.5 ± 1.3	17.2 ± 7.5	0.13	0.31	0.52
With TCDD mass > 7,000 ng (avg mass = 11,898 ± 4,048 ng)	7 (15)	3 (3)	1.9 ± 1.0^c^	16.1 ± 5.0	0.10	0.26	0.27
Chloracne severity comparisons							
No chloracne	8 (18)	12 (30)	2.7 ± 1.4	14.7 ± 7.1	0.14	0.54	0.53
Low grade (grade 1 or 2)	7 (23)	2 (6)	2.2 ± 1.2^d^	20.1 ± 7.8	0.12	−0.24	0.68
High grade (grade 3 or 4)	10 (35)	6 (15)	1.9 ± 1.1^d^,^e^	16.3 ± 6.2	0.14	−0.41	0.65
All chloracne (grades 1–4)	17 (58)	8 (21)	2.0 ± 1.1^d^,^e^	17.7 ± 7.0	0.13	−0.26	0.68
Sex comparisons							
Male	0	20 (51)	2.5 ± 1.2	16.1 ± 6.8	0.12	0.59	0.46
Female	25 (75)	0	2.1 ± 1.3	17.0 ± 7.3	0.13	−0.04	0.53
Selected age group comparisons							
< Age 12	13 (27)	9 (16)	1.5 ± 0.8	9.4 ± 1.6	−0.02	1.70	0.002
< Age 18	19 (40)	15 (33)	1.6 ± 0.9	11.4 ± 2.9	0.03	1.30	0.01
≥Age 18	22 (34)	18 (18)	3.2 ± 1.2^f^	24.0 ± 4.1	0.18	−1.16	0.41
Selected latency from exposure comparisons							
< 15 years latency	21 (52)	14 (33)	1.7 ± 0.9	12.9 ± 4.6	0.05	−1.10	0.06
> 15 years latency	23 (23)	18 (18)	3.5 ± 1.1^g^	24.6 ± 4.3	0.14	−0.02	0.31

Abbreviations: avg, average; conc, concentration. *n*_female_ and *n*_male_ denote the number of female and male subjects included in the subset, and *n*_values_ denotes the number of valid half-life values included.

a Significantly different mean value compared to the BMI < 20 group mean at *p* < 0.05 using the *Z*-statistic or Student’s *t*-test.

b Significantly different mean value compared to the BMI < 25 group mean at *p* < 0.05 using the *Z*-statistic or Student’s *t*-test.

c Significantly different mean value compared to the paired group mean at *p* < 0.05 using the *Z*-statistic test.

d Significantly different mean value compared to the Nonchloracne group mean at *p* < 0.05 using the *Z*-statistic test.

e Significantly different mean value compared to the Nonchloracne group mean at *p* < 0.05 using Student’s *t*-test.

f Significantly different mean value compared to the < Age 12 and < Age 18 group mean at *p* < 0.05 using the *Z*-statistic or Student’s *t*-test.

g Significantly different mean value compared to the < 15 years latency group mean at *p* < 0.05 using the *Z*-statistic or Student’s *t*-test.
