# Supplementary material for: Age- and Concentration-Dependent Elimination Half-Life of 2,3,7,8-Tetrachlorodibenzo-p-dioxin in Seveso Children
Source: Environ Health Perspect. 2006 Jul 6;114(10):1596–602. doi: 10.1289/ehp.8884 (PMC1626409; doi:10.1289/ehp.8884)
Supplement: Supplemental Figures and Tables [file ehp0114-001596s1.pdf]

Supplemental Material:

Methods

Age-related variables evaluated as fixed effects include age at time of initial sampling (initial age), age at time of subsequent sampling (age), and the time period following initial sampling (latency). Body composition variables evaluated as fixed effects included BFM and BMI. TCDD body burden variables evaluated as fixed effects included the natural logarithm of TCDD concentration measured in the initial peak sample (ln initial sample), the natural logarithm of TCDD concentration measured in subsequent samples (ln concentration), the natural logarithm of TCDD mass estimated for initial sample (ln initial mass), and the natural logarithm of the TCDD mass estimated for subsequent samples (ln mass). The log transformed body burden variables were used because scatter plots of these log transformed variables versus half life displayed a linear trend.

An evaluation of the intercorrelations among these variables was performed to identify variables that are highly correlated with each other in order to avoid including them in the same model. The different combinations of these three variable types were included in separate models and evaluated relative to each other using the Akaike Information Criterion (AIC) (Akaike 1974). The AIC is a statistical measure for comparing different models and is dependent on both the goodness-of-fit and the number of independent variables included in the model. The best model based on this criterion is the one with the lowest AIC value.

Results and Discussion

To determine which of the various independent variables to include in the mixed regression model, the pair-wise correlations between these variables were estimated (Table A-1). As expected, the highest correlations are between age and latency ($r = 0.80$), BFM and BMI ($r = 0.92$), and log concentration and log mass ($r = 0.92$). Initial age is significantly correlated ($r = 0.56$; $p < 0.05$) with age but is not significantly correlated with latency. Age appears to be an independent variable that includes both the initial age and latency. In addition, BFM and BMI are significantly correlated with age and latency with a moderate correlation coefficient of 0.47.

The different groups of variables were evaluated by starting with a baseline model that included initial age, latency, BFM, and ln initial TCDD concentration, and modifying the variables associated with each category—age, body composition, or body burden—in order to determine the best variable for each category (Tables A-2 to A-5).

For the age variables, there was not much difference between the baseline model and the model that included age instead of initial age and latency (Table A-2). The AIC values for both models were close with the AIC for the baseline model equal to 231 and the AIC for the alternative model equal to 244. For both models, the only coefficient that was not significantly different than zero was BFM ($p = 0.260$ for baseline; $p = 0.686$ for alternative). Because age has more general applicability in pharmacokinetic models of child body burden compared to initial age and latency, age will be used in the final regression model.

For the body composition variables, there was no difference between the baseline model and the alternative model that included BMI instead of BFM (Table A-3). For both models, the body composition variables were not significantly different than zero and the AIC values were identical. Because of the correlation of BFM and BMI to the age variables, an alternative model that included only BFM, and ln initial TCDD concentration was evaluated. In this alternative model the coefficient for BFM was significantly different than zero (p < 0.0001) and this model had a fairly high AIC value of 334. This indicates that the intercorrelation between the age variables and the body composition variables causes multiple colinearity problems in models for which both are included. This is not surprising because body composition for children changes with age. Because the AIC value for the model with BFM but not age is much higher than those with age variables, any final model will include age rather than BFM or BMI.

For the body burden (concentration) variables, the use of ln TCDD concentration versus ln TCDD mass makes little difference in the regression model (Table A-4). For both variables, the coefficient for the body burden variable is significantly greater than 0 ($p \leq 0.001$) and the AIC values are similar. However, comparing the use of initial ln TCDD concentration versus ln TCDD concentration indicates that the choice of concentration variable has a significant impact on the model. For the baseline model that includes initial ln TCDD, the model predicts that higher initial TCDD concentrations are associated with lower half lives, which is consistent with trends seen in the previous analyses. However, for the model that includes ln TCDD concentration, the model predicts that higher TCDD concentrations are associated with higher half lives, which is

inconsistent with trends seen in the previous analyses. Although ln initial concentration appeared to be the most appropriate body burden metric to include in the final model on the basis of preceding mixed regression studies, the initial concentration is not a variable that can be easily generalized and applied to any pharmacokinetic models of child TCDD body burden.

Due to above problems, an alternative analysis was developed based on the observed concentration-dependent transition point in the half life data (on a linear scale) at a TCDD concentration approximately 700 ppt. A categorical variable for TCDD concentration (TCDD concentration category) was defined: 0 for TCDD concentrations ≤ 700 ppt, and 1 for TCDD concentrations ≥ 700 ppt. In addition, this categorical variable was crossed with age to create a variable called TCDD concentration category × age to evaluate any effects of the categorical variable on the regression coefficient for age.

References

Akaike, H. 1974. A new look at the statistical model identification. IEEE Transactions on Automatic Control 19:716-723.

Table A-1. Correlation coefficients among independent variables evaluated in mixed regression model.

	**Initial age**[a]	**Age**[b]	**Latency**	**BFM**	**BMI**	**Ln Initial TCDD conc.**	**Ln TCDD conc.**
Age[b]	0.56[c]	—					
Latency[d]	–0.055	0.80[c]	—				
Body fat mass[e]	0.13	0.47[c]	0.47[c]	—			
BMI[f]	0.052	0.44[c]	0.49[c]	0.92[c]	—		
Ln initial TCDD concentration[g]	–0.20	–0.062	0.083	–0.069	–0.15	—	
Ln TCDD concentration[h]	0.23	–0.28	–0.51[c]	–0.35	–0.42[c]	0.42[c]	—
Ln TCDD mass in body[i]	0.36[c]	–0.042	–0.32[c]	0.006	–0.064	0.41[c]	0.92[c]

a Age at the time of initial sampling.
b Age at the time of subsequent sampling.
c Correlation coefficient statistically significant after Bonferroni correction at a 95% confidence level ($p < 0.05$).
d Latency is defined as the time since the initial sampling.
e body fat mass
f body mass index
g Natural log of the TCDD concentration measured at the initial sampling.
h Natural log of the TCDD concentration measured at the subsequent sampling.
i Natural log of the TCDD mass calculated from the body fat mass and the TCDD concentration measured at the subsequent sampling.

Table A-2. Summary of mixed regression models used to evaluate the age term.[a]

#	Subject effect[b]	Initial age	Latency	Age	BFM	Ln initial TCDD conc.	AIC[c]
**1**	3.7 ± 0.4 $p < 0.0001$	0.07 ± 0.02 $p < 0.0001$	0.15 ± 0.01 $p < 0.0001$	—	–0.011 ± 0.01 $p = 0.26$	–0.39 ± 0.04 $p < 0.0001$	231
**2**	3.0 ± 0.4 $p < 0.0001$	—	—	0.13 ± 0.01 $p < 0.0001$	–0.004 ± 0.01 $p = 0.67$	–0.35 ± 0.05 $p < 0.0001$	244

Each box contains the estimate of the mixed regression coefficient of the variable ± the standard error and followed by the p value for the null hypothesis that the coefficient is not significantly different than zero. P values less than 0.05 indicate that the coefficient is significantly different than zero at a 95% confidence level.

— Variable not included in the model.

a The complete data set as described in the Methods section was used for each of these three models.

b The subject effect is described by the random intercept term in the mixed regression model.

c The Akaike Information Criterion (AIC) is a statistical measure for comparing different models that is dependent on both the goodness-of-fit of the model to the data and the number of independent variables included in the model (Akaike 1974). The best model is the one with the lowest AIC value.

Table A-3. Summary of mixed regression models used to evaluate the body composition term.[a]

#	Subject effect [b]	Initial age	Latency	BFM	BMI	Ln initial TCDD conc.	AIC [c]
1	3.7 ± 0.37 $p < 0.0001$	0.07 ± 0.02 $p < 0.0001$	0.15 ± 0.01 $p < 0.0001$	–0.01± 0.01 $p = 0.26$	—	–0.39 ± 0.04 $p < 0.0001$	231
3	3.8 ± 0.52 $p < 0.0001$	0.07 ± 0.02 $p < 0.0001$	0.15 ± 0.01 $p < 0.0001$	—	–0.01 ± 0.02 $p = 0.53$	–0.39 ± 0.04 $p < 0.0001$	232
4	4.5 ± 0.58 $p < 0.0001$	—	—	0.07 ± 0.01 $p < 0.0001$	—	–0.36 ± 0.07 $p < 0.0001$	334

Each box contains the estimate of the mixed regression coefficient of the variable ± the standard error and followed by the p value for the null hypothesis that the coefficient is not significantly different than zero. P values less than 0.05 indicate that the coefficient is significantly different than zero at a 95% confidence level.

— Variable not included in the model.

a The complete data set as described in the Methods section was used for each of these three models.

b The subject effect is described by the random intercept term in the mixed regression model.

c The Akaike Information Criterion (AIC) is a statistical measure for comparing different models that is dependent on both the goodness-of-fit of the model to the data and the number of independent variables included in the model (Akaike 1974). The best model is the one with the lowest AIC value.

Table A-4. Summary of mixed regression models used to evaluate the concentration term.[a]

#	Subject effect[b]	Initial age	Latency	BFM	Ln TCDD conc.	Ln TCDD mass	Ln initial TCDD conc.	AIC[c]
**1**	3.7 ± 0.37 $p < 0.0001$	0.07 ± 0.02 $p < 0.0001$	0.15 ± 0.01 $p < .0001$	–0.011 ± 0.01 $p = 0.26$	—	—	–0.39 ± 0.04 $p < 0.0001$	231
**5**	–1.1 ± 0.53 $p = 0.042$	0.08 ± 0.04 $p = 0.019$	0.18 ± 0.02 $p < 0.0001$	–0.003 ± 0.01 $p = 0.82$	0.27 ± 0.07 $p < .0001$	—	—	272
**6**	–1.3 ± 0.60 $p = 0.035$	0.08 ± 0.04 $p = 0.024$	0.17 ± 0.01 $p < 0.0001$	–0.013 ± 0.01 $p = 0.28$	—	0.24 ± 0.07 $p = 0.001$	—	273

Each box contains the estimate of the mixed regression coefficient of the variable ± the standard error and followed by the *p* value for the null hypothesis that the coefficient is not significantly different than zero. *P* values less than 0.05 indicate that the coefficient is significantly different from zero at a 95% confidence level.

— Variable not included in the model.

a The complete data set as described in the Methods section was used for each of these three models.

b The subject effect is described by the random intercept term in the mixed regression model.

c The Akaike Information Criterion (AIC) is a statistical measure for comparing different models that is dependent on both the goodness-of-fit of the model to the data and the number of independent variables included in the model (Akaike 1974). The best model is the one with the lowest AIC value.
